# Antiretroviral prophylaxis to prevent post-natal transmission of HIV through breastfeeding

**Published:** 2008-08-15

**Authors:** Louise Kuhn

**Affiliations:** 1 Department of Epidemiology, Mailman School of Public Health & Gertrude H. Sergievsky Center, College of Physicians and Surgeons, Columbia University, New York.

**Keywords:** nevirapine, zidovudine, sub-saharan Africa, breastfeeding, PMTCT

Recently published results provide important new “proof of principle” that providing antiretroviral drugs to infants of HIV-infected mothers while breastfeeding can reduce postnatal HIV transmission.[1, 2] These are very encouraging results as they demonstrate that HIV infections can be reduced while preserving benefits of breastfeeding for these vulnerable infants. In the first study called SWEN (Six Week Extended-Dose Nevirapine) over 2000 mother-baby pairs in Ethiopia, India and Uganda were randomized to either single-dose nevirapine or to an extended regimen that included the standard maternal and newborn dose of nevirapine plus an extended course of nevirapine to the infant starting at 8 days of life and continuing daily to 42 days. After excluding those who were already infected at birth, the mother-to-child HIV transmission rate at 6 weeks was 5.3% in the single-dose nevirapine group and 2.5% in the six week extended-dose nevirapine group.[1] In the second study called PEPI (Post Exposure Prophylaxis of Infants) over 3000 mother-baby pairs in Malawi were randomized to either the control group that used single-dose nevirapine plus a week of zidovudine or to one of the two intervention groups: (1) daily nevirapine from 1 week to 14 weeks or (2) daily nevirapine and zidovudine from 1 week to 14 weeks. After excluding those who were already infected at birth, the mother-to-child HIV transmission rate at 14 weeks was 8.4% in the control group, and 2.8% and 2.8% in the extended prophylaxis arm using only nevirapine or using both nevirapine and zidovudine, respectively. [2]

We should not be surprised if the response of policy-markers and communities to these results is one of puzzlement. How should these new results be translated into new policies? Should extended nevirapine prophylaxis for 6 or 14 weeks become a routine part of prevention of mother-to-child HIV transmission (PMTCT) programs in sub-Saharan Africa? In both studies there was an attenuation of the benefit of the extended nevirapine prophylaxis when children were tested some months after the prophylaxis had ended. In the SWEN study, transmission rates (excluding the birth infections) at 6 months were 9.0% in the control group and 6.9% in the extended prophylaxis group – a difference that was no longer statistically significant. [1] In PEPI, transmission rates (excluding the birth infections) at 9 months were 10.6% in the control group and 5.2% and 6.4% in the extended prophylaxis groups using only nevirapine or using both nevirapine and zidovudine, respectively. Although the benefit of extended prophylaxis remained statistically significant in PEPI, the magnitude of the benefit declined from a 3-fold decrease in transmission at 14 weeks to a 2-fold decrease at 9 months.[2] Although there had been some hope that the transmission-reducing action of nevirapine would continue after consumption of the drug had stopped, it appeared that later breastfeeding-acquired infections accrued at the same rate regardless of whether or not the extended prophylaxis had been offered.

A naïve, but dangerous, conclusion from these observations might be that breastfeeding should end at the same time as extended prophylaxis ends, in other words, weaning should be encouraged at either 6 or 14 weeks. Highly disturbing findings in both of these studies are the extremely short durations of breastfeeding reported. In the SWEN study, only 32% of infants were still breastfeeding at 6 months. In PEPI, 90% were still breastfeeding at 6 months, but by 9 months almost 70% were not.[1, 2] Scientific studies of this magnitude take time to complete with the rigor required, and it should be noted that the SWEN study enrollment began in February 2001, PEPI in April 2004. At the time these studies were designed there was less appreciation of the risks of prematurely truncating the duration of breastfeeding. It was only in October 2006 that the World Health Organization (WHO) revised their guidelines to clearly encourage exclusive breastfeeding for the first 6 months of life as an option for HIV-infected mothers. It was also only at this time that WHO revised their guidelines to clearly state that early weaning should only be considered if there are affordable, feasible, acceptable, safe and sustainable (AFASS) replacement options for the infant or young child.[3] The latter recommendation, while a veiled one, is a recognition of the weak health service infrastructures, poor environmental conditions and high rates of other severe childhood infections in most communities in sub-Saharan Africa that make early weaning a dangerous proposition. We have recently published our results from the Zambia Exclusive Breastfeeding Study (ZEBS) in which we observed no benefit of in terms of HIV-free survival of early cessation of breastfeeding at 4 months compared to standard practice of breastfeeding to an average of 16 months. [4] The net benefit in terms of transmissions averted by stopping breastfeeding earlier than usual was cancelled out by the mortality caused by the absence of breastfeeding after 4 months. The ZEBS results are consistent with findings from Botswana that observed that HIV-free survival was not improved by formula feeding from birth compared to breastfeeding to about 6 months.[5] Weaning at 6 or even 14 weeks is therefore much too early!

Might extended prophylaxis not simply be continued throughout the full duration of breastfeeding whatever its length? Both the SWEN and PEPI investigators encourage further study of this as an option and the ZEBS results clearly indicate the need to investigate interventions that allow the usual duration of breastfeeding (i.e. up to 18 months or greater) to occur. Theoretically, infant prophylaxis throughout breastfeeding should be safe and effective in at least reducing, if not entirely eliminating, transmission. But I would argue against implementation without evaluation. Too often reasonable inferences drawn from observational research are not borne out in clinical trials. A multi-site study is underway to investigate extended prophylaxis to 6 months.[6] Let us hope that investigation of extended prophylaxis to the full course of breastfeeding will not have to await small monthly increments until full duration of breastfeeding is reached. In the meantime, much could be gained by implementing 14 weeks of extended nevirapine prophylaxis as long as the program were not coupled with explicit or implicit messages to terminate breastfeeding as soon as feasible after the end of prophylaxis.

Infant prophylaxis is an attractive approach to prevention of postnatal transmission as relatively simple, inexpensive and safe regimens can be used, and the risks of these interventions are accrued in the same population (infants) who stand to benefit. However, infant prophylaxis has one serious drawback in that it neglects the needs of the mother. Maternal prophylaxis, in contrast, stands to benefit both mother and child. One program that provided effective triple antiretroviral therapy to all pregnant women in Mozambique regardless of their clinical conditions or CD4 counts claims to have observed a very low transmission rate [7] but these data are difficult to interpret since the program was not set up to investigate the effects of this intervention on postnatal transmission. Others have raised concerns about short and long-term adverse effects of placing HIV-infected women on antiretroviral drugs before they are required on clinical grounds. However, for those HIV-infected pregnant women who do require effective antiretroviral therapy on clinical and CD4 count grounds, there is no dispute about the urgency of their receiving antiretroviral therapy. No-one disagrees that providing access to effective antiretroviral therapy for these women is an essential component of any comprehensive HIV prevention and treatment program as well as an essential component of any PMTCT program. Antiretroviral therapy can save the young mother’s life, can substantially reduce HIV-related morbidity, and has been shown (in observational studied only) to reduce the risk of transmitting the virus during pregnancy and delivery. This is a classic win-win public health intervention: providing a beautiful example of positive treatment-prevention synergy. Poor coordination between HIV care and PMTCT programs often, unfortunately, compromises optimal implementation of this logical recommendation. Effective maternal treatment is expected to reduce postnatal transmission but data demonstrating this are sparse. Investigation of this question poses challenges since treatment cannot be withheld from this group of women.

As public health professionals, we welcome new findings that provide evidence of new interventions that have the capacity to improve our programs. As scientists, we have to stay attuned to what we still do not know, and have the foresight to design new studies to begin to address those gaps. Although antiretroviral drugs have a crucial role to play in prevention of mother-to-child HIV transmission through breastfeeding, we still have much to learn about how best to use these drugs taking into account the social and biological context of maternal and child health in the many disadvantaged sub-Saharan African settings where the HIV epidemic predominates.
